# Complexity-Based Measures of Postural Sway during Walking at Different Speeds and Durations Using Multiscale Entropy

**DOI:** 10.3390/e21111128

**Published:** 2019-11-16

**Authors:** Ben-Yi Liau, Fu-Lien Wu, Chi-Wen Lung, Xueyan Zhang, Xiaoling Wang, Yih-Kuen Jan

**Affiliations:** 1 Department of Biomedical Engineering, Hung Kuang University, Taichung 443, Taiwan; byliau@sunrise.hk.edu.tw; 2 Rehabilitation Engineering Lab, Department of Kinesiology and Community Health, University of Illinois at Urbana-Champaign, Champaign, IL 61820, USA; fulienwu@illinois.edu (F.-L.W.); mrkx88@126.com (X.Z.); xw39@illinois.edu (X.W.); 3 Department of Creative Product Design, Asia University, Taichung 443, Taiwan; 4 Beijing Advanced Innovation Center for Biomedical Engineering, Beihang University, Beijing 10000, China

**Keywords:** center of pressure, complexity, falls, multiscale entropy, postural control

## Abstract

Participation in various physical activities requires successful postural control in response to the changes in position of our body. It is important to assess postural control for early detection of falls and foot injuries. Walking at various speeds and for various durations is essential in daily physical activities. The purpose of this study was to evaluate the changes in complexity of the center of pressure (COP) during walking at different speeds and for different durations. In this study, a total of 12 participants were recruited for walking at two speeds (slow at 3 km/h and moderate at 6 km/h) for two durations (10 and 20 min). An insole-type plantar pressure measurement system was used to measure and calculate COP as participants walked on a treadmill. Multiscale entropy (MSE) was used to quantify the complexity of COP. Our results showed that the complexity of COP significantly decreased (*p* < 0.05) after 20 min of walking (complexity index, CI = −3.51) compared to 10 min of walking (CI = −3.20) while walking at 3 km/h, but not at 6 km/h. Our results also showed that the complexity index of COP indicated a significant difference (*p* < 0.05) between walking at speeds of 3 km/h (CI = −3.2) and 6 km/h (CI = −3.6) at the walking duration of 10 min, but not at 20 min. This study demonstrated an interaction between walking speeds and walking durations on the complexity of COP.

## 1. Introduction

Postural control is a complex process based on continuous and interactive information between our sensorimotor system and the environment [1,2,3,4]. Participation in various physical activities requires successful postural control in response to changes in the position of our body [5,6]. People with sensorimotor impairments due to disease or aging lose their postural control, which results in falls and foot injury. It is important for clinicians to assess the postural control function changes in at-risk populations to prevent fall-related injuries. 

Various measures have been used to assess postural control functions, including center of mass, center of gravity, and center of pressure (COP) [1,2,3,4]. Among these measures, the analysis of COP has been widely used for quantitative assessments of postural control due to its easy measurement. COP is the point of the ground reaction force (GRF) vector acting on the plantar foot, which starts from heel strike, moves forward, and ends near the toes at toe-off. COP is a signal that reflects ankle torque and COP trajectory progression is time-varying and represents the muscle force for body stabilization [7]. Common COP-based indexes include the COP trajectory, the front/back maximum offset, and the left/right maximum offset. Research studies show that the shorter the trajectory and the smaller the offset, the better the balance [8,9,10].

Recently, researchers have shown that these traditional measurements may not fully characterize the changes of posture control associated with aging or pathological conditions [11,12]. This new evidence shows that traditional linear characteristics of COP trajectories may not be sensitive to detecting balance changes and the use of nonlinear analysis (e.g., complexity) may be more sensitive for detecting pathological changes of postural control and balance [11,12]. The complexity in a physiological system reflects the adaptability of the system to various stimuli [13,14,15,16]. Such complexity assessments have been widely introduced in various pathophysiological assessments for better diagnoses and assessments. In postural control, complexity of COP refers to a person’s ability to adapt to various postural needs and a higher complexity of COP may imply a better capability for postural adaptability. Researchers have used entropy to study the complexity changes of COP in pathological conditions [17,18,19,20,21]. Purkayastha et al. suggested that the nonlinear complexity of COP might be more sensitive to detect insufficient postural control abilities, compared to linear variables [22]. Moreover, complexity-related variables of COP showed a better reliability than the COP velocity [23]. However, the COP data of most studies were derived from static double leg standing or single leg standing. The tasks adopted by these studies might not reflect the characteristics of postural control in daily life. Only two studies, conducted by Mei et al., compared the complexity of COP displacement, velocity, and acceleration, extracted from walking with sample entropy, concluding that sample entropy can be a possible evaluation method to identify different foot types [24,25]. Yet, they collected the plantar pressure data from a pressure plate system rather than an in-shoe plantar pressure system, which required participants to perform many walking trials to record enough data points.

Multiscale entropy (MSE) is a method to quantify complexity (i.e., regularity) of a time series (e.g., COP time series) at multiple scales [26,27,28]. MSE has been proposed to fully quantify complexity in the COP trajectories [12]. Although the complexity index of COP has shown some promising effects in evaluating static postural control or in identifying potential pathologies, the entropy used in these studies may not reveal different scales of complexity [29]. This might be the reason that the use of entropy in assessing COP is still not consistent and inconclusive. Understanding comprehensive postural control mechanism during walking by analyzing the MSE of COP may provide researchers and clinicians with valuable information to develop an appropriate postural control assessment or to design an optimal walking training program. 

Participation in physical activities and activities of daily living involve various walking speeds and durations. However, it is unclear how engaging in activities with various walking speeds and durations affect postural sway and stability. This is particularly important in populations at risk for falls and foot injuries [30]. Although traditional COP analysis provides the information of COP displacement and velocity, the traditional analysis may lose the information of dynamical complexity due to the non-stationary nature of COP [31]. Therefore, we performed a nonlinear analysis of COP by multiscale entropy to observe changes in complexity during walking at different speeds and for different durations. The purpose of this study was to assess the complexity of COP during walking at different speeds and for different durations using MSE. To the best of our knowledge, this is the first study to explore the changes of complexity of COP during walking at different speeds and for different durations. 

## 2. Methods

### 2.1. Subjects

Healthy subjects between 18 and 45 years of age were recruited from the university and nearby community. Exclusion criteria were active foot ulcers, diabetes, vascular diseases, hypertension, the inability to walk for 20 min independently, the inability to walking on the treadmill independently, or the use of vasoactive medications. Each subject signed the informed consent approved by the University of Illinois at Urbana-Champaign Institutional Review Board (#19225) before the screening and experimental procedures. 

### 2.2. Experimental Procedures

All examinations were performed in the Rehabilitation Engineering Laboratory at the University of Illinois at Urbana-Champaign. Room temperature was fixed at 24 ± 2 °C. All subjects relaxed in the supine position for at least 30 min prior to testing to stabilize the baseline blood flow level and acclimate themselves to the room temperature. Two speeds (slow walking at 3 km/h and moderate walking at 6 km/h) [32], and two durations (10 min and 20 min) were tested in this study. All participants were asked to walk with an appropriate pair of shoes at a speed of 3 km/h on a treadmill at the first week, and 6 km/h at the second week. For each week, participants were randomly assigned into the 10 min or 20 min walking duration first, and the other duration later, with a balanced crossover design. For avoiding carryover effects and muscle fatigue, participants were allowed to rest for at least 20 min between 10 min and 20 min walking trials. 

### 2.3. Center of Pressure Measurements

An F-scan system (Tekscan, South Boston, MA) was used to measure the plantar pressure data of the right foot in standardized shoes [33] during walking on the treadmill. Each F-scan in-shoe sensor contains 960 sensing pixels (sensels). The sensor was placed between the subject’s sock and the insole of the shoe. A subject wore the sensors inside the shoes for 3–5 min of walking before the walking experiment. Each time, the sensor was calibrated according to the manufacturer’s instructions. Data were sampled at 200 Hz. Center of pressure data were extracted from the Tekscan software. 

### 2.4. Multiscale Entropy Analysis

The definition of “entropy” in thermodynamics is a measure of unavailability in a closed system, which describes the degree of the disorder state in a system [34]. Several algorithms based on the concept of entropy have been applied to measure the complexity of physiological signals. Multiscale entropy (MSE) is one of the methods developed by Costa et al. [13,28,34]. MSE uses the algorithm of “sample entropy” to estimate the regularity in different time scales, based on the approximate entropy, to assess the complexity degree [13,28,34].

First, a one-dimensional discrete time series {*x_1_,x_2_,….x_n_*} is reconstructed by the scale factor “*τ*” to be coarse-grained time series with different time scales. Each element of $y_{j}^{\left(\tau \right)}$ is according to Equation (1), as follows:

(1) $$y_{j}^{\left(\tau \right)}=\frac{1}{\tau }\sum_{i=\left(j-1\right)\tau +1}^{j\tau }x_{i},1\leq j\leq \frac{N}{\tau }.$$

By the coarse-grained procedure, sample entropy (SE) can be estimated by the scale factor “*τ*”. SE is defined by Equation (2), as follows:

(2) $$\mathrm{SE}\left(m,\gamma ,\tau \right)=-\log\frac{A_{\tau }}{B_{\tau }},$$

where *m* is the a template vector of length, *A* is the number of template vector pairs having $d\left[x_{m+1}\left(i\right),x_{m+1}\left(j\right)\right]<\gamma$, and *B* is the number of template vector pairs having $d\left[x_{m}\left(i\right),x_{m}\left(j\right)\right]<\gamma$.

The complexity index (CI) can be estimated from SE by Equation (3), which is the summation of SE from scale factor 1 to the maximum.

(3) $$\mathrm{CI}=\sum_{i=1}^{\tau }\mathrm{SE}\left(i\right).$$

Regarding the required data length for MSE (sensitivity), it has been suggested that 200 data points per window are needed to elicit consistent SE values, though some studies used 600 data points at the longest time scale [29]. Previous research showed that the shortest coarse-grained time series should be 300 data points [30]. In this study, we have almost 1,000 data points that are sufficient for MSE analyses. Two-way repeated measures ANOVA was used to examine the interaction between the walking speeds and walking durations on the complexity of COP. Paired t tests were used to examine the statistical significance. Cohen’s effective size was also calculated to estimate the effect size [35]. The significance level was set as 0.05. All analyses were performed using MATLAB R2017b (MathWorks, Inc., Natick, MA, USA). 

## 3. Results

Twelve healthy subjects (5 men, 7 women) were recruited in this study. The demographic data were as follows (mean ± standard deviation): Age, 25.7 ± 5.5 years (range 21–42 years); height, 171.3 ± 8.5 cm (range 157–188 cm); weight, 64.2 ± 13.5 kg (range 50–93 kg); and BMI, 22.04 ± 2.93 kg/m^2^ (range 19.05–28.39 kg/m^2^). Regarding BMI, 10 subjects were in the healthy range and 2 subjects were in the overweight range; none were in the obese range. 

**Figure 1:**
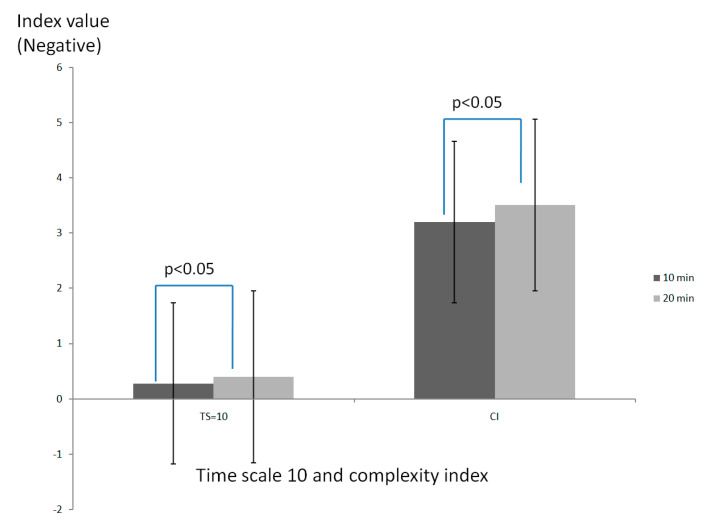
Comparison of complexity of center of pressure (COP) between 10 min and 20 min walking durations while walking at a speed of 3 km/h.

Figure 1 shows that time scale 10 of MSE and the complexity index of COP significantly decreased (*p* < 0.05) after 20 min of walking (complexity index, CI= −3.51) compared to 10 min of walking (CI = −3.20) at the waking speed of 3 km/h, but not at the walking speed of 6 km/h. 

**Figure 2:**
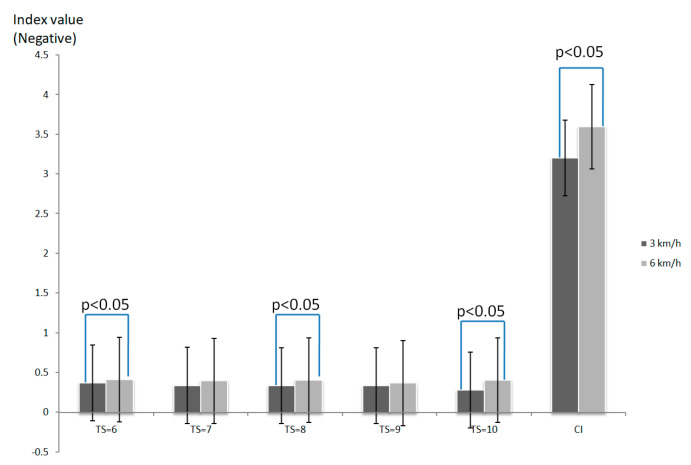
Comparison of complexity of COP between 3 km/h and 6 km/h walking speeds while walking for 10 min.

Figure 2 shows the complexity index of COP indicated a significant difference (*p* < 0.05) between walking at speeds of 3 km/h (CI= −3.2) and 6 km/h (CI = −3.6) at the walking duration of 10 min, but not at the walking duration of 20 min. The *x*-axis is the time scale and complexity index. The *y*-axis is the index value. In this comparison, several time scales and complexity indexes revealed significant differences.

Two-way repeated measures ANOVA was performed to assess the interaction effect between the walking speed and walking duration on the CI of COP. The results showed the interaction effect (*p*-value < 0.01). 

The size effect was calculated using Cohen’s d algorithm. It showed that the value of Cohen’s d estimated by the different walking durations while walking at 3 km/h was larger than 0.8 (large effect size). In addition, the value of Cohen’s d, estimated by the different walking speeds for the 10 min walking duration, was larger than 0.9 (large effect size). 

**Figure 3:**
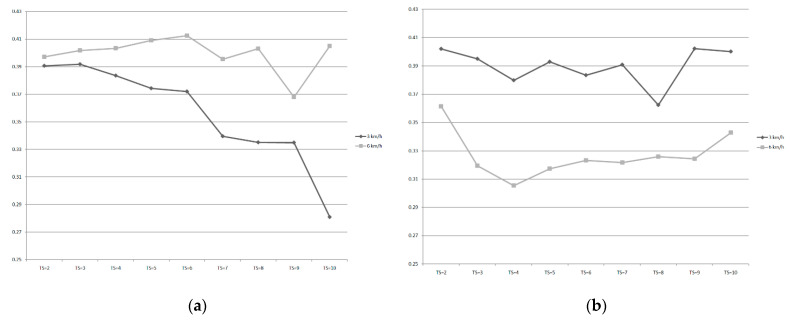
(**a**) Trend of complexity of COP between 3 km/h and 6 km/h in each time scale of walking speeds while walking for 10 min. (**b**) Trend of complexity of the COP between 3 km/h and 6 km/h in each time scale while walking for 20 min.

Figure 3 shows the trend of complexity of the COP between 3 km/h and 6 km/h walking speeds while walking for 10 and 20 min. It can be found that the trend of complexity of COP while walking for 10 min is separated more obviously with increasing the time scale until time scale 10, even in the contrasting trend, which is significantly different. However, while walking for 20 min, the effect of walking speed on the complexity of COP is mild due to there being no significant differences in each time scale.

## 4. Discussion 

The main finding of this study is that we demonstrated that both walking speed and walking duration factors may significantly affect the complexity of COP. Our results also showed that complexity of COP significantly decreased after 20 min of walking compared to 10 min of walking, and while walking at 3 km/h, but not at 6 km/h. Our results also showed that the complexity of COP indicated a significant difference between walking at speeds of 3 km/h and 6 km/h at the walking duration of 10 min, but not at 20 min. 

Our study demonstrated that there is no significant difference between CI during 10 and 20 min walking at 6 km/h, which is significantly different from walking at 3 km/h. Few studies have investigated how people change their gait patterns or adjust their posture after various durations of walking [36,37]. Thomas and colleagues studied static postural sway every 5-min interval over 35 min of walking at three different speeds, suggesting that physically active young adults demonstrated the ability of static postural adaptation at the beginning of the fast walking and then maintained the ability until the end of the task [37]. Stolwijk et al. measured plantar pressure data from people who completed a 4-day marching event, indicating that after marching people tend to walk with less roll-off movement and more heal loading. The authors argued that altered gait patterns after prolonged walking might be from lower limb muscle fatigue [36]. In this study, the non-significant difference at 6 km/h between 10 min and 20 min walking durations could be due to an easier adaptation to the walking speed at 6 km/h. 

In contrast to 3 km/h, our results showed that slow walking at 3 km/h for 20 min significantly decreased the complexity compared to 10 min. This could be due to lower leg muscle fatigue. The finding indicates that walking at a slower speed (3 km/h) may easily decrease the complexity of COP compared to walking at a higher speed (6 km/h). This may imply that slower walking speed may not improve postural control. Our finding is supported by the literature, which showed that higher walking speed and shorter step length may improve stability [38,39]. Older people, usually considered to have poor postural control, tend to adopt the strategy of a slower speed (slower than preferred walking speed) and a shorter step length than young people during walking [40,41,42]. Short step length and low walking speed, which were exhibited by the elder people, however, may cause opposite effects on postural stability during walking. Future studies need to investigate the effects of walking speeds on postural control by different variables in order to to broaden the understanding about the differences between fallers and non-fallers. 

A study conducted by McClymont et al. showed that the mean square error, one of the variability measures, of plantar pressures significantly increased when walking faster, while the coefficient of variation, another standard variability measure, was not related to walking speed [43]. Lu et al. performed a series of studies to determine the effects of different gait speeds controlled by treadmill or participants on the inclination angle of center of mass and the COP, suggesting that the minimum value of the range of the frontal inclination angle was detected at the preferred walking speed, compared to that at lower or higher speeds, either in over-ground walking or in treadmill walking [44,45]. These findings indicate that people might control their posture with the least effort during walking at their preferred speed. Thus, that older people choose a non-preferred, slower walking speed may not help them improve balance, but rather it may cause them to lose balance and postural control ability. Moreover, people could adjust their posture quickly within 5 min of fast walking; thus, this may be the reason that we could not observe any difference between CI during 10 and 20 min of walking at 6 km/h.

The use of MSE on COP may provide a new window to assess postural control. The MSE approach could evaluate how disorders or diseases affect postural oscillations via time series scales that reflect the time domain dynamics of the complex network of our postural control system. Our results are consistent with previous reports of complexity studies. With the same time scale factor and complexity index, a longer walking duration would decrease the complexity index (CI) significantly (*p* < 0.05). This is consistent with clinical observations that walking for a longer time could decrease balance capacity; in our case, 20 min compared to 10 min of walking. With the walking velocity factor, only the results of 3 km/h vs. 6 km/h after 10 min of walking showed a significant difference (*p* < 0.05). As walking faster could reduce the CI value of COP, it can be speculated that walking faster would also reduce the walking balance. Traditional measures may be useful in patients with major impairments in postural control, while complexity analysis may be more sensitive for early detection of postural deficits in various pathological conditions, as well as in the elderly. 

Various methods have been developed to identify people at risks for falls and foot injuries. Among them, COP analysis is an easy to use method in various settings. COP, or parameters associated with plantar pressures, has been used as an evaluation tool to quantify the postural control function. Previous research showed that peak plantar pressures of most plantar regions generally increased with increased walking speed [46,47,48,49,50]. Elevated plantar pressures may result in abnormally cumulated stress over the plantar soft tissue. COP, an important index calculated from plantar pressure data, is able to assess postural control and to predict the risk of falls [51,52,53,54,55]. Previous researchers have examined the trajectories, velocity, or variability of COP to evaluate the dynamic postural control during walking at different speeds. Chiu et al. found that the COP velocity significantly increased with increased walking speed, whereas there was no significant difference in the COP progression angle between different walking speeds [56]. In this study, we used MSE to analyze COP while waking at different speeds and for different durations and demonstrated that this method could be useful to complement current methods for assessing postural control by providing information about the complexity status of postural control. 

In this study, the age of participants ranged from 21 to 42 years. According to the literature [57,58], people aged above 60 years demonstrate significant changes in COP progression and variability from the younger population. Age between 18 and 60 years is not a significant factor affecting COP. Thus, the age factor may not significantly affect our results. Regarding the range of body mass index in this study, 10 subjects were in the healthy range and 2 subjects were in the overweight range; none were in the obese range. Obesity (BMI above 30 kg/m^2^) may increase postural sway during quiet standing [59,60,61,62] or gait initiation [63]. We believe that the body weight and BMI factors may not significantly affect the current results.

Our findings have several clinical implications. Patients with impaired sensorimotor function tend to walk at a slower speed to improve postural control. In this study, we demonstrated that walking duration may be a more significant factor for postural control, compared to walking speeds. This finding suggests that people at risk for falls and foot injuries may need to avoid walking for a longer duration, for which it may be the time (walking for a long time) that causes them to fall. Investigating the COP alteration when individuals perform walking at different speeds and for different durations for various activities is essential to understand the postural control mechanism so that clinical practitioners can design an appropriate exercise program for those who have balance control problems.

There are limitations of this study. First, this study only recruited healthy subjects, which limits its generalization to patients at risks for falls and foot injury. However, our study demonstrated that, even in healthy subjects, the MSE of COP revealed significant changes between walking for 10 and 20 min. Our method could be applied to study pathological changes in patients. Second, a study design with longer walking durations and faster walking speeds should be investigated to further understand how walking speeds and durations affect the complexity of COP. Third, the COP data were calculated using the in-shoe pressure measurement system. The accuracy of the in-shoe pressure measurement system is not as accurate as the force plate system. However, our in-shoe system provides the advantage of less restricted data collection requirements for activities.

## 5. Conclusions

Using MSE analysis of COP during walking at different speeds and durations, we found that both walking speed and walking duration factors significantly affect the complexity of COP. The MSE analysis of COP may provide new information to evaluate postural control in people at risk for falls and foot injuries.
